# Sustained delivery of chABC improves functional recovery after a spine injury

**DOI:** 10.1186/s12868-022-00734-8

**Published:** 2022-10-28

**Authors:** Atousa Sharifi, Ali Zandieh, Zahra Behroozi, Michael R. Hamblin, Sara Mayahi, Mahmoud Yousefifard, Fatemeh Ramezani

**Affiliations:** 1 Department of Medical Nanotechnology, Faculty of Advanced Technologies in Medicine, Iran University of Medical Sciences, Tehran, Iran; 2 Department of Physiology, School of Medicine, Iran University of Medical Sciences, Tehran, Iran; 3 Laser Research Centre, Faculty of Health Science, University of Johannesburg, Doornfontein, 2028 South Africa; 4 Radiation Biology Research Center, Iran University of Medical Sciences, Tehran, Iran; 5 Physiology Research Center, Iran University of Medical Sciences, Tehran, Iran; 6 Physiology Research Center, Institute of Neuropharmacology, Kerman University of Medical Sciences, Kerman, Iran

**Keywords:** chABC, Scaffold, Hydrogel, Spinal cord injury: animal model

## Abstract

**Introduction:**

Chondroitinase ABC (chABC) is an enzyme could improve regeneration and thereby improving functional recovery of spinal cord injury (SCI) in rodent models. Degradation of the active enzyme and diffusion away from the lesion are the causes of using hydrogels as a scaffold to deliver the chABC into the lesion site. In this meta-analysis, we investigated the effects of chABC embedded in a scaffold or hydrogel on the functional recovery after SCI.

**Method:**

Databases were searched based on keywords related to chABC and spinal cord injury (SCI). Primary and secondary screening was performed to narrow down study objectives and inclusion criteria, and finally the data were included in the meta-analysis. The standard mean difference of the score of the functional recovery that measured by Basso, Beattie, Bresnahan (BBB) test after SCI was used to analyze the results of the reported studies. Subgroup analysis was performed based on SCI model, severity of SCI, transplantation type, and the follow-up time. Quality control of articles was also specified.

**Results:**

The results showed that embedding chABC within the scaffold increased significantly the efficiency of functional recovery after SCI in animal models (SMD = 1.95; 95% CI 0.71–3.2; p = 0.002) in 9 studies. SCI model, severity of SCI, injury location, transplantation type, and the follow-up time did not affect the overall results and in all cases scaffold effect could not be ignored. However, due to the small number of studies, this result is not conclusive and more studies are needed.

**Conclusion:**

The results could pave the way for the use of chABC embedded in the scaffold for the treatment of SCI and show that this method of administration is superior to chABC injection alone.

## Introduction

Spinal cord injury (SCI) is caused by damage to the spinal cord that causes is associated with temporary or permanent impairment in neurological function [1, 2]. SCI is divided into two types: traumatic and non-traumatic. Traumatic SCI occurs because of motor vehicle accidents, falls, sports injuries, violent assault, etc. While non-traumatic SCI is produced by diseases such as tumors, infections, etc. [3, 4]. Traumatic SCI often lead to devastating loss of sensory and motor function, and despite therapeutic methods which have shown positive results in the research phase [5–10] so far there is no drug treatments that can restore function consistently in affected patients Although there is some spontaneous regenerative responses that occurs following SCI, this is often insufficient for significant improvement [11]. Several factors, including inappropriate immune responses, glial scar development, and lack of adequate neurotrophic support after SCI may be the cause of this lack of regenerative ability [12–15]. The glial scar and the cystic cavities that are formed over the long term tend to decrease remyelination and inhibit axonal regrowth [16]. As a result, the healing of the spinal cord is very poor compared to other types of injured tissue [17, 18].

Several experiments have shown that at the site of the lesion, chondroitin sulfate proteoglycans (CSPGs) are secreted in increasing quantities by the stimulated astrocytes and oligodendrocytes. These CSPGs subsequently inhibit the axonal regeneration that might occur after SCI [19–21]. The glial scar is also a physical barrier to axonal regeneration. CSPGs are inhibitory molecules that accumulate at the site of the lesion and prevent neurite extension, neuronal growth, and neuroplasticity both in vitro and in the injured CNS in-vivo [12, 22–25]. CSPGs is an extracellular matrix component that contains two common structural parts; one is the protein core (NG2 protein), and the attached long glycosaminoglycan (GAG) polysaccharide chains [26–29].

Chondroitinase ABC (chABC) is an enzyme that can break the sulfated GAG chains on the CSPGs which are a main element of the glial scar [30–33]. Degradation of the glial scar has been shown to stimulate axonal growth, leading to improved function in some models of SCI in rodents [17, 34–36]. chABC also degrades the CSPGs that form perineuronal nets, thereby enhancing axonal sprouting and plasticity [37–39]. However, the use of chABC in animal models has problems that have caused the effect of chABC to be moderately reported in a meta-analysis study based on the results of 34 preclinical studies [40].

One major issue is that the administration of chABC by injection, by using an intrathecal catheter, or pumping through a cannula cannot guarantee sufficient sustained concentrations of drug at the injury site [23, 30]. Moreover, because chABC is a protein it remains active for only 3–5 days at 37 °C [41–43].

One approach to prevent premature degradation of the enzyme and to increase its efficacy is to use a variety of scaffolds that can be loaded with chABC for sustained release [30, 43, 44]. These scaffolds are often hydrogels that possess low toxicity and are also biocompatible and biodegradable [45–47]. In various studies, chABC has been loaded into hydrogel scaffolds, and the effect on the treatment of SCI has been investigated in animal models. Some of these scaffolds have included PLGA nanoparticles [48], PLLA microspheres incorporated within a chitosan scaffold [49], other types of hydrogel such as methylcellulose hydrogel [30], agarose hydrogel-microtube scaffold system [41], cross-linked methylcellulose (XMC) hydrogel, collagen scaffold [43], or alginate electrospun scaffold [50].

In this meta-analysis, we compared the effects of chABC when loaded inside a scaffold with untreated animals, on functional improvements after SCI in animal models.

## Material and methods

The guidelines of Preferred Reporting Items for Systematic Reviews and Meta-Analyses (PRISMA) were used for this study. The review protocol for this study has not been published.

### Search strategy

Using keyword groups related to chABC and spinal cord injury, listed in Table 1, a systematic search with unlimited language limitation for published articles up to June 30, 2022, was performed in the databases of SCOPUS, EMBASE, MEDLINE, and Web of Science. A manual exploration was also accompanied to find further articles.

**Table 1:** Search strategies used in PUBMED

PUBMED	(“Chondroitin-Sulfate-ABC Endolyase” [MESH] or “Chondroitin Sulfate ABC Endolyase” [MESH] or “Chondroitinase ABC” [MESH] or “Chondroitin-Sulfate-ABC Endolyase” [tiab] or “Chondroitin Sulfate ABC Endolyase” [tiab] or “Chondroitinase ABC” [tiab] or “chondroitinaseABC” [tiab] or “Chondroitin ABC eliminase” [tiab] or “Chondroitin ABC lyase” [tiab] or “Chondroitin sulfate ABC endoeliminase” [tiab] or “Chondroitin sulfate ABC endolyase” [tiab] or “Chondroitin sulfate ABC lyase” [tiab] or “Chondroitinase”[tiab] or “Chondroitinase ABC”[tiab] or “ChS ABC lyase” [tiab] or “ChS ABC lyase I” [tiab]) and ("spinal cord injury"[MeSH] OR "spinal cord contusion"[MeSH] OR "spinal cord hemisection"[MeSH] OR "spinal cord transsection"[MeSH] OR "cervical spine injury"[MeSH] OR "spinal cord injury"[tiab] OR "spinal cord contusion"[tiab] OR "spinal cord hemisection"[tiab] OR "spinal cord transsection"[tiab] OR "cervical spine injury"[tiab] OR "Spinal compression"[tiab] OR "spinal cord trauma"[tiab] OR "trauma, spinal cord"[tiab] OR "injured spinal cord"[tiab] OR "spinal cord injured"[tiab] OR "spinal cord injuries"[tiab] OR "nerve transection"[tiab])

### Study selection, eligibility, and rejection standards

Duplicate articles were removed and then the two reviewers reviewed the articles separately by reading the titles, abstracts, and complete texts (if the titles and abstracts did not cover necessary information). Disagreements over selection of studies were resolved through discussions between them.

Only studies were included for analysis that reported the effect of chABC embedded in any type of scaffold, using in-vivo experiments in animals with SCI, and the result using the BBB test to measure functional recovery was compared with the untreated SCI group.

Exclusion criteria were: review articles, articles that did not use chABC embedded in scaffolding as intervention, studies that did not compare the treatment group with the control group.

### Data extraction and quality assessment

Both assessors independently extracted the following information from the articles: animal features, species, gender, weight, age, sample size, studied organ, duration of exposure, study time, type of transplantation of chABC (implant or injection), chABC concentration, vehicle name and the score of BBB test which represent the functional recovery after SCI. Analysis was performed by calculation of standard mean differences (SMD) of the mean of BBB test and the reported standard deviation for each group. The risk of bias assessment of articles was achieved based on the study of Hassannejad et al. [51].

### Statistical analysis

For data analysis STATA 14.0 statistical software was used. Data as mean and standard deviation were presented. The effect size was calculated with a 95% confidence interval (95% CI). The fixed-effect model was used, and if the heterogeneity was more than 50%, the random-effect model was used. Using Egger’s precision-weighted linear regression method, the existence of publication bias was explored, and the results were available in funnel plots. Significant level in all analyses was considered p < 0.05.

## Results

### Included studies

**Fig. 1:**
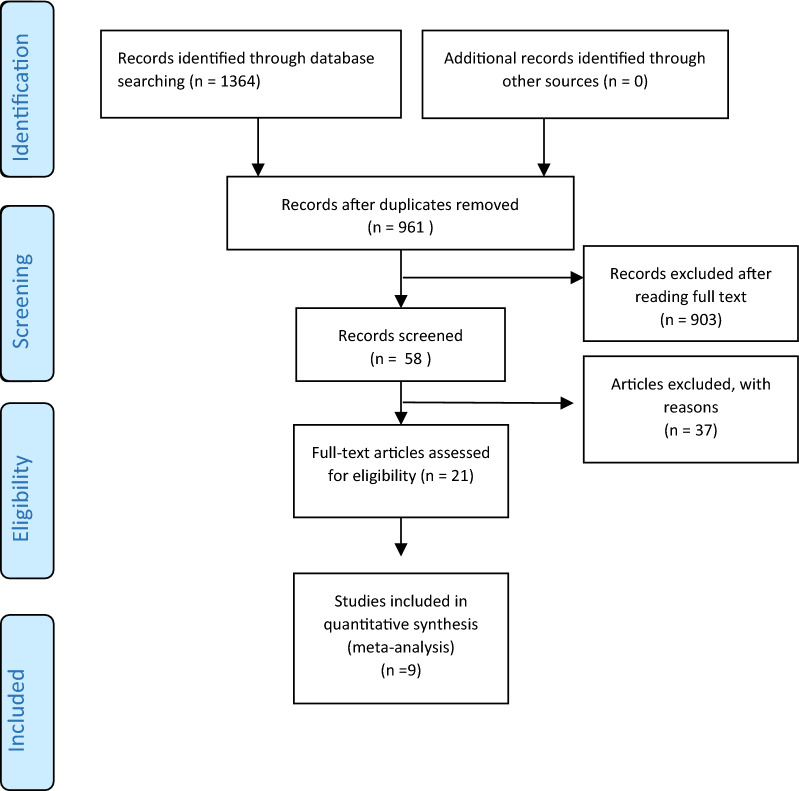
PRISMA flow diagram for systematic reviews displaying database search details, the number of abstracts, and the full texts included in the study

1364 articles were initially acquired from a general search of the records. When removing identical articles, 961 articles were carefully chosen based on evaluation of the titles and abstract. After evaluating the full text, 9 articles finally were used for the meta-analysis. Figure 1 displays the flow chart of the search procedure and the choice of articles.

### Data extraction

9 studies look at the effect of chABC embedded in a scaffold on animal locomotion after SCI. The data gained from these articles is prepared in Table 2. All 9 articles used a rat model. Two studies were performed on the T1-T2 location and the others were performed on T7–T10. All 9 articles used the BBB test to measure the locomotor activity of the animals. Therefore, a meta-analysis was performed to explore the effect of chABC embedded in a scaffold on the BBB test results.

**Table 2:** Data of the studies included in the systematic review and meta-analysis

Refs.	Gender; species; strain; weight or age	Model of injury; location of injury; severity	Numbers: sham; chABC alone; scaffold chABC	Interval time from injury to treatment	chABC concentration	Transplantation type (implant or injection)	Nanoscaffold or hydrogel	FU (week)
Azizi et al. [48]	Male; rat; Wistar; 275 ± 25 g	Contusion; T10; severe	5; NA; 6	1 week	%1 W/W	Injection	PLGA nanoparticles	8
Führmann et al. [53]	Female; rat; SD; 300 g	Compression; T1-2; moderate	n ≥ 9; n ≥ 9; n ≥ 9	chABC immediately; cell injection 1 week later	5 μl	Injection	Methylcellulose (XMC) hydrogel	9
Cholas et al. [52]	Female; rat; Lewis; 150–175	Hemiresection; T8-9; severe	6; NA; 8	0 day	25 µL of a 10-Units/mL chABC	Implant	EDAC-cross-linked collagen scaffold	4
Pakulska et al. [30]	Female; rat; SD; 200–250 g	Compression; T1-2; moderate	11; 11; 11	Immediately after injury	10 µg	Injection	Methylcellulose hydrogel	8
Xia et al. [54]	Female; rat; Wistar; 200–230 g	Hemitransection; T7–T9; moderate	6; 6; 6	During the procedure	NR	Implant	Poly(propylene carbonate) microfibers scaffold	4
Zhang et al. [55]	Female; rat; SD; 200–250 g/2–3 mo	Segmental transversal injury; T10; severe	8; 8; 8	After surgery	0.25 U	Injection	PLGA delayed-release microspheres	10
Pan et al. [56]	Female; rat; Wistar; 250 g	Transection; T10; severe	8; 12; 12	Immediately after injury	10 U/mL	Implant	Poly(glycerol sebacate)	12
Ni et al. [57]	Female; rat; Wistar; 200–230 g	Hemisection; T7–T9; severe	6; 6; 6	Immediately after injury	%1 W/W	Implant	Poly (propylene carbonate)-chitosan micron fibers scaffold	4
Raspa et al. [33]	Female; rat; SD; 250–275 g	Compression; T9—T10, moderate	8; 8; 8	4 weeks after injury	0.1 µ/ml	Injection	FAQ self assembled peptide	6 weeks after injury

### Quality control

The calculation of the risk of bias indicated low risk of bias in all articles in the following categories: species, strain, genetic background, age/weight, and number of animals per group, description of control, method of group allocation, objective tissue, use of suitable tests, and description of statistical analysis.

2 articles displayed a great risk of bias regarding the blinding of the assessor, 6 articles displayed a high risk of bias regarding randomization, 2 articles displayed a high risk of bias in terms of the definition of the experimental unit, 2 articles showed a high risk of bias in terms of description of the animal facility, 7 articles showed a high risk of bias in term of regulations and ethics, and 8 articles showed a high risk of bias in term of the reasons to exclude animals from the study (Table 3).

**Table 3:** Bias items in all included studies

Author	1	2	3	4	5	6	7	8	9	10	11	12	13	14	15	16
M. Azizi	Y	Y	Y	Y	Y	Y	Y	Y	Y	Y	Y	Y	Y	Y	N	N
T. Führmann	Y	Y	Y	Y	Y	Y	Y	Y	Y	Y	N	Y	Y	Y	Y	N
R. Cholas	Y	Y	Y	Y	Y	Y	Y	Y	Y	Y	N	Y	Y	Y	N	N
M. Pakulska	Y	Y	Y	Y	Y	Y	Y	Y	Y	N	N	N	Y	Y	N	N
T. Xia	Y	Y	Y	Y	Y	Y	Y	Y	Y	Y	N	Y	Y	Y	N	N
Y. Zhang	Y	Y	Y	Y	Y	Y	Y	Y	Y	N	Y	Y	Y	N	Y	Y
Q. Pang	Y	Y	Y	Y	Y	Y	Y	Y	Y	Y	Y	N	Y	N	N	N
Shilei Ni	Y	Y	Y	Y	Y	Y	Y	Y	Y	Y	N	Y	Y	Y	N	N
Andrea Raspa	Y	Y	Y	Y	Y	Y	Y	Y	Y	Y	N	N	Y	Y	N	N

1. Species, 2. Strain, 3. Age/weight, 4. Genetic background, 5. Number of animals per group, 6. Definition of control, 7. Method of allocation to treatments, 8. Target tissue, 9. Using appropriate tests, 10. Blinding the assessor, 11. Randomization 12. Definition of the experimental unit (individual animal/animals in one cage), 13. Description of statistical analysis, 14. Animal facility, 15. Regulation and ethics, 16. Description of the reasons to exclude animals during the study (attrition)

### Results of meta-analysis

**Fig. 2:**
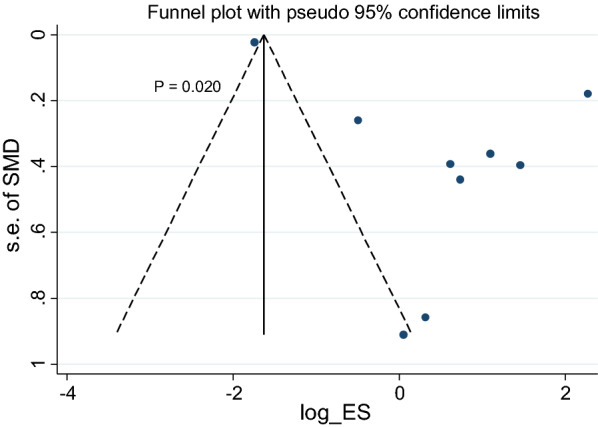
Funnel plot of articles on the effects of chABC embedded in a scaffold on the motor function recovery in animal models of SCI. *SMD* standardized mean difference

In the current study, publication bias was observed in 9 studies describing the effects of a scaffold embedded with chABC on the motor function recovery test of animals after SCI (p = 0.02) (Fig. 2).

**Fig. 3:**
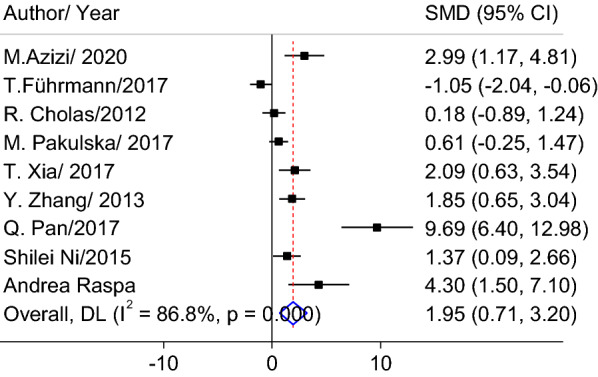
Forest plot of the effects of chABC embedded in a scaffold on behavioral tests after SCI compared to non-treated animals. *CI* confidence interval, *SMD* standardized mean difference

The results of the meta-analysis showed that embedding chABC into a scaffold had a significant effect on the recovery of motor function (SMD = 1.95; 95% CI 0.71–3.2; p = 0.002) (Fig. 3). The results also showed that there was a significant heterogeneity between the studies of motor function recovery (I^2^ = 86.8%, p < 0.001).

In the subgroup analysis (Table 4), it was found that the following factors relevant to the SCI model had no statistical effect on the outcome. These were contusion-compression (SMD = 1.4; 95% CI:− 0.52 to 3.31; p = 0.15) or transection-hemisection (SMD = 2.458; 95% CI: 0.688–4.228; p = 0.006); the follow-up time, which was < 8 weeks (SMD = 1.55; 95% CI: 0.572–2.527; p = 0.002) or ≥ 8 weeks (SMD = 3.11; 95% CI: − 0.89 to 7.1; p = 0.128); severity of injury which was severe (SMD = 2.694; 95% CI: 0.793–4.595; p = 0.005) or moderate (SMD = 1.146; 95% CI: − 0.567 to 2.858; p = 0.19); or the transplantation method which was by injection (SMD = 1.45; 95% CI − 0.093 to 2.997; p = 0.0.065) or by implantation (SMD = 2.801; 95% CI 0.399–5.204; p = 0.022). None of these factors had any influence on the effects of scaffold embedded chABC on the motor function recovery after SCI (Table 4), and in all the sub groups the scaffold embedded chABC had a significant effect compared to the untreated animals.

**Table 4:** Subgroup analysis of treatment effect of scaffold embedded chABC on motor function recovery after SCI compared to non-treated animals

Subgroup	Number of experiments	Heterogeneity (p value)	SMD (95% CI)			p value
SCI model						
Contusion-compression	4	87.3% (< 0.0001)	1.4	− 0.52	3.31	0.15
Transection–hemisection	5	87% (< 0.0001)	2.458	0.688	4.228	0.006
Severity of SCI						
Severe	5	87.8% (< 0.0001)	2.694	0.793	4.595	0.005
Moderate	4	85.7% (0.001)	1.146	− 0.567	2.858	0.19
Transplantation type						
Injection	5	85.7% (< 0.0001)	1.45	− 0.093	2.997	0.065
Implant	4	90.0% (< 0.0001)	2.801	0.399	5.204	0.022
Follow-up time						
< 8 weeks	6	67.2% (0.0091)	1.55	0.572	2.527	0.002
≥ 8 weeks	3	95.5% (< 0.0000)	3.11	− 0.89	7.1	0.128
Overall	9	86.8% (< 0.0001)	1.95	0.708	3.198	0.002

*CI* confidence interval, *SCI* spinal cord injury, *SMD* standardized mean difference

## Discussion

In this article, the effect of the chABC enzyme embedded in a scaffold on the treatment of SCI was systematically investigated. The results showed that the chABC embedded in a scaffold could significantly improve the functional recovery compared to untreated animals.

Many pre-clinical articles have examined the effects of chABC injection on the improvement of functional recovery after SCI [58–62]. The results of a meta-analysis showed that the enzyme chABC injected alone had a moderate effect on the functional recovery post-SCI (SMD = 0.90; 95% CI 0.61 to 1.20; p < 0.001) [40].

The results of the present study that analyzed all articles that employed chABC embedded in a hydrogel or a scaffold, showed that its effect on the functional recovery post-SCI was stronger and more significant than chABC alone. Based on the result, none of the factors involved in the SCI model, SCI severity, location of the injury, type of transplant, follow-up time, influenced on the therapeutic effect that according to the small number of studies makes it impossible to accept this result with certainty. Treatment of a damaged spinal cord with chABC is designed to attenuate the inhibitory effects of CSPGs that accumulate in the lesion site, and is expected to improve overall functional recovery. Injection of the free enzyme is the most common method to administer chABC, but the disadvantage of the injection at the lesion site, is that the injected chABC is subject to rapid degradation by the host enzyme and body temperature [63, 64], and also spreads away from the site of injury [41]. There are major limitations to the path of clinical treatment through direct injection of chABC; ChABC after 1 h (h) of incubation at 37 °C fails 50% of its enzymatic activity. Therefore, to circumvent this barrier, several injections of ChABC, infusion through mini-pumps/catheters or use of scaffolds have been used to provide stable continuous local delivery of fresh chABC in vivo [33]. The result of this meta-analysis showed when the chABC is locally delivered by embedding it into a scaffold which allows slow-release, this approach can maintain higher levels of the bioactive enzyme at the lesion site for a longer time and thus increase the beneficial effect on neuronal regeneration and functional recovery [60, 65].

Although our analysis was based on 9 articles, and the number of articles could be seen as one of the limitations, nevertheless the results can pave the way for the use of scaffold-embedded chABC to treat SCI and suggests this route of administration is superior to injection of chABC alone.

## Data Availability

The findings of this study are derived from data that are available upon request from the responsible author (FR).
